# Comparisons of Laboratory and On-Road Type-Approval Cycles with Idling Emissions. Implications for Periodical Technical Inspection (PTI) Sensors

**DOI:** 10.3390/s20205790

**Published:** 2020-10-13

**Authors:** Barouch Giechaskiel, Tero Lähde, Ricardo Suarez-Bertoa, Victor Valverde, Michael Clairotte

**Affiliations:** European Commission, Joint Research Centre (JRC), 21027 Ispra, Italy; tero.lahde@ec.europa.eu (T.L.); ricardo.suarez-bertoa@ec.europa.eu (R.S.-B.); victor.valverde-morales@ec.europa.eu (V.V.); michael.clairotte@ec.europa.eu (M.C.)

**Keywords:** vehicle emissions, particle number, periodical technical inspection, idle, roadworthiness

## Abstract

For the type approval of compression ignition (diesel) and gasoline direct injection vehicles, a particle number (PN) limit of 6 × 10^11^ p/km is applicable. Diesel vehicles in circulation need to pass a periodical technical inspection (PTI) test, typically every two years, after the first four years of circulation. However, often the applicable smoke tests or on-board diagnostic (OBD) fault checks cannot identify malfunctions of the diesel particulate filters (DPFs). There are also serious concerns that a few high emitters are responsible for the majority of the emissions. For these reasons, a new PTI procedure at idle run with PN systems is under investigation. The correlations between type approval cycles and idle emissions are limited, especially for positive (spark) ignition vehicles. In this study the type approval PN emissions of 32 compression ignition and 56 spark ignition vehicles were compared to their idle PN concentrations from laboratory and on-road tests. The results confirmed that the idle test is applicable for diesel vehicles. The scatter for the spark ignition vehicles was much larger. Nevertheless, the proposed limit for diesel vehicles was also shown to be applicable for these vehicles. The technical specifications of the PTI sensors based on these findings were also discussed.

## 1. Introduction

Air pollution, especially particulate matter (PM), has significant impacts on the health of the European population. It was estimated that in 2016 the mass of PM below 2.5 micron was responsible for about 412,000 premature deaths in Europe [1]. The road transport contributed to 11% of total PM mass primary emissions in the 28 countries of the European Union in 2017 [1]. In addition to the PM mass, there is also a concern about the contribution of traffic originated ultrafine (<0.1 μm) particles to the detriment effect on human health [2], as the road traffic is the major ultrafine particle number source in most cities [3]. Both the mass and number of traffic originated particles have shown significant reductions in the last 15–20 years [4], and it is estimated that they will further decrease in Europe until 2030 [5]. The concentration reductions can be attributed to policies (e.g., more stringent Euro emission standards), traffic management, and fleet restrictions (e.g., low emission zones) [4]. Policies focused mainly on vehicle exhaust emissions, but as the levels have decreased, the contribution of non-exhaust emissions (from brakes and tires) can contribute at similar levels [6].

In the European Union (EU) the particle number (PN) and PM mass vehicles exhaust emissions have to respect some limits defined in the regulations. The type approval of a vehicle family requires that some limits depending on the date of registration are respected (e.g., Euro 5). The procedure includes measurement of the emissions of a representative vehicle during a pre-defined driving cycle in the laboratory under well controlled ambient conditions. The type approval cycle in Europe was the NEDC (New European Driving Cycle), which was replaced by the WLTC (Worldwide harmonized Light vehicles Test Cycle) in 2017 with Euro 6c (Commission Regulation EU 2017/1151). Furthermore, in 2017 a Real-Driving Emissions (RDE) test on the road was introduced in the type-approval procedure including a Not-To-Exceed (NTE) limit for PN with Euro 6d-temp (Commission Regulation EU 2017/1154) [7]. Further provisions ensure the conformity of production (i.e., checking sample vehicles from the production line) and in-service conformity (checking vehicles already circulating in the streets). The laboratory PN instruments are based on the Particle Measurement Programme (PMP) group recommendations [8]. The on-road tests are conducted with portable emissions measurement systems (PEMS) [9]. Limits are applicable for compression ignition (diesel) vehicles since 2011 (Euro 5b) and gasoline direct injection (GDI) vehicles since 2014 (Euro 6b). The current PN limit of solid (nonvolatile) particles is 6 × 10^11^ p/km. However, for the first three years (2014–2017), a limit of 6 × 10^12^ p/km could be applied to new GDI vehicles upon request of the manufacturer [10].

The roadworthiness regulation (Directive 2014/45/EU, which repealed Directive 2009/40/EC) ensures that all circulating vehicles are kept in a safe and environmentally acceptable condition. It requires appropriate measures to prevent adverse manipulation of, or tampering with, vehicle parts and components that could have a negative bearing on required safety and environmental characteristics of the vehicle. In order to check the emissions of a vehicle, a periodical technical inspection (PTI) test is required for all circulating vehicles, typically every two years, after the first four years of circulation. Exhaust gas smoke emissions of diesel vehicles are measured with opacimeters during free acceleration (no load from idle up to cut-off speed) with gear lever in neutral and clutch engaged. Alternatively, the reading of OBD (on-board diagnostics) can be used, if available. However, in a study with 400 vehicles, 6% of them had high smoke emissions and none of them had any DPF (Diesel Particulate Filter) fault codes at the OBD reading [11], indicating that OBD systems are not always well designed to detect DPF failures. Similarly, in another study the OBD was unable to detect any DPF faults [12]. The opacity test is also obsolete for todays’ vehicles because the opacity limit is quite high. A study showed that all vehicles with and without DPF could pass the current limits (1.5 m^−1^ and 0.7 m^−1^ for Euro 5 and Euro 6, respectively) [13]. Another study showed that even a 100% damaged DPF resulted in emissions well below the PTI limit, although 0.5% and 5% damage ratios resulted in values exceeding the PN and PM mass limits in type approval tests [14]. Lowering the opacity limits has the challenge that it is already close to the detection limit of the method (0.3 m^−1^). Furthermore, a study showed that all diesel vehicles (Euro 5) with or without DPF had smoke emissions <0.5 m^−1^ [15]. Recent studies concluded that, instead of using opacimeters for the determination of smoke emissions, laser light scattering sensors could be used: they were sufficiently accurate and stable, and had the necessary dynamic response characteristics and resolution for testing modern vehicles [12,14]. However, a practicable calibration procedure needs to be defined for light scattering sensors. Furthermore, concerns have been raised for their high dependence on the particle size and the resulting low sensitivity for small nanoparticles [16].

Because current PTI procedures cannot detect such high emitters of PM, their DPFs are not repaired or replaced and the contribution of these high emitters could increase the average fleet emissions [17], even by a factor 30 [13]. Some studies showed that a small percentage of high polluting vehicles can account for the majority of the emissions [18]. Depending on the pollutant, <10% of the fleet can contribute 30–85% of the emissions [16,19,20,21]. Various inspections found that 5−15% of the inspected vehicles were high emitters with damaged or removed DPFs [13,22]. The fail rate increased with mileage: from 3% (<50,000 km) to 25% (>150,000 km). Identifying and removing from the road high emitters (result of damaged or tampered particulate filters) should result in an important reduction of the contribution of on-road transport to the total particle emissions.

The VERT (Verification of Emission Reduction Technologies) Association, Swiss, German, and Dutch governmental organizations, metrological institutes, scientists, and equipment manufacturers established an informal new periodical technical inspection (NPTI) technical working group. The working group is developing methodologies for both DPFs and NO_x_ aftertreatment systems [23]. In 2017 a White Paper summarized the proposal with a PN test at low idle for diesel vehicles [24]. A report from TNO (Netherlands Organisation for Applied Scientific Research) in the same year gave more details [25]. Low idle was chosen as it is simple: only the average of at least 15 s is needed, after a stabilization time of at least 15 s. Snap accelerations followed by low idle speed operation were excluded because they resulted in a non-defined engine behavior. The accelerations affected the activation of the EGR (exhaust gas recirculation) in a non-predictable and non-repeatable way. The Netherlands introduced a PTI regulation in November 2019, applicable to Euro 5b and later diesel vehicles, with a PN limit of 2.5 × 10^5^ p/cm^3^, to be met at low idle [26]. The foreseen implementation year is 2021, when measurement sensors will be available to the inspection centers. Belgium and Germany are considering adopting a similar regulation.

Although the work with compression ignition vehicles is at a good level, the studies on positive (spark) ignition vehicles are limited, and without clear conclusions whether the procedure used on diesel vehicles is applicable. Furthermore, there is lack of correlation data of type approval emission tests and idle concentrations. The objective of this study is to present comparisons of type approval cycles and idle concentrations for both compression ignition and positive ignition vehicles and to suggest thresholds based on the experimental results.

Section 2 describes the experimental setup and explains the analysis that was followed with an example. Emissions on the complete laboratory and on-road type-approval cycles are compared to laboratory hot and cold idle concentrations in Section 3. The impact of the DPF soot load on the measured idle concentrations is presented in Section 4 along with the implications of these results for the PTI instruments.

## 2. Materials and Methods 

### 2.1. Experimental Setup

**Figure 1:**
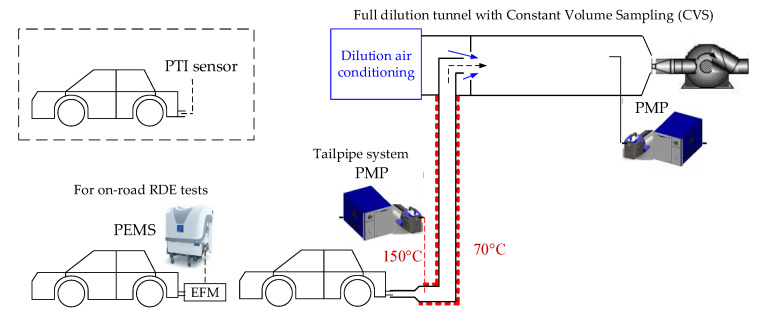
Experimental setup of this study in the laboratory or on the road. In the laboratory the particle number systems (PMP) where connected to the tailpipe and/or to the full dilution tunnel (CVS). On the road particle number PEMS were used. The typical new PTI setup, used in some studies of the literature, is also shown on the top left corner of the figure in dashed lines. CVS = Constant Volume Sampling; EFM = Exhaust Flow Meter; PEMS = Portable Emissions Measurement System; PMP = Particle Measurement Programme; PTI= Periodical Technical Inspection; RDE = Real-Driving Emissions.

The typical experimental setup of this study is presented in Figure 1. The type-approval laboratory measurements were conducted from a tunnel where the whole exhaust gas was diluted with filtered air and using constant volume sampling (CVS). 

The PN system was based on the Particle Measurement Programme (PMP) recommendations and the regulation technical requirements (2017/1151). In all cases it was the AVL (Graz, Austria) Advanced Particle Counter (APC 489) with an evaporation tube at 350 °C, and a Condensation particle Counter (CPC) with 50% detection efficiency at 23 nm [27]. The vehicle followed a pre-defined test cycle (NEDC or WLTC) and the emissions were determined in p/km (see, e.g., [28] for calculation details). In order to have comparable conditions with PTI measurements that are sampling from the tailpipe, a second PMP system identical or similar to the system at the dilution tunnel was connected to the tailpipe. The idle solid particle concentrations were determined during cold start (<300 s) or with hot engine in p/cm^3^.

In many cases on road tests were conducted using a PEMS (Portable Emissions Measurement System) from AVL (MOVE). The PN-PEMS used a catalytic stripper at 300 °C and measured solid particle number concentration with a cut-off of 23 nm by means of a diffusion charger sensor. Euro 6b and older vehicles were tested with prototype PEMS, because the AVL MOVE was not available then: the Nanomet 3 (from Testo, Lenzkirch, Germany; formerly Matter Engineering) which had an evaporation tube at 300 °C and a diffusion charger to count solid particles or the modified NPET (from Horiba, Kyoto, Japan) which had a catalytic stripper at 350 °C and a CPC with 50% detection efficiency at 23 nm. Details about the PEMS instruments can be found elsewhere [9].

### 2.2. Vehicles

Table 1 summarizes the number of vehicles and the tests that were available: WLTC or NEDC type approval laboratory emissions and hot idle concentrations were available for all cases. In total, data from 32 diesel vehicles (6 of them without DPF, and 6 from the literature), 31 GDI vehicles (4 with GPF, 2 from the literature), 18 PFI vehicles and 7 LPG or CNG vehicles were found. RDE emissions and cold idle concentrations were available only for a fraction of the vehicles. For some older and relatively high emitting vehicles, the idle concentrations were determined from the CVS because no tailpipe measurements were available. Details will be given in the next section.

The data were taken from the following studies:

- TNO: PN emissions at low idle speed and NEDC tests of 4 different diesel vehicles with (cracked) DPF or variable bypass [22,25].
- JRC PTI study: PN emissions at low idle and WLTC of 4 different vehicles (two diesel, one GDI, one GDI with GPF), one of them (diesel) bypassing the DPF [29].
- PN-PEMS studies: Laboratory studies that compared PN-PEMS with PMP systems at the tailpipe or the dilution tunnel [30,31,32,33]. From the studies that investigated the 10 nm PEMS, only the 23 nm information was used [34,35].
- Tailpipe studies: Laboratory studies that compared PMP systems at the tailpipe and the dilution tunnel [28,36].
- Emissions monitoring: Laboratory and on-road assessment of various vehicles [37,38,39,40,41,42,43,44,45,46].
- Unpublished data: Internal data of older vehicles (non DPF diesel vehicles, or GDIs emitting >6 × 10^12^ p/km).

No other studies were identified in the literature that reported both idle and type approval emissions.

### 2.3. Analysis and Calculations

**Figure 2:**
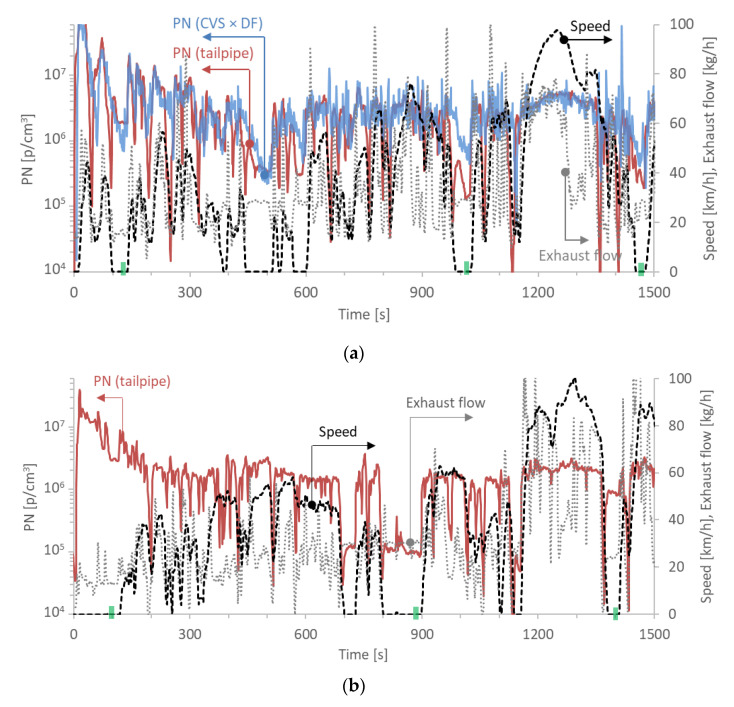
Real time particle number (PN) emissions, exhaust flow rate and speed trace for the first 1500 s of: (**a**) a WLTC (upper panel) and (**b**) a RDE test (lower panel) of a Gasoline Direct Injection (GDI) vehicle. Arrows indicate the appropriate y-axis. The green indexes at the time x-axis show the period that idle concentrations were calculated. CVS = Constant Volume Sampling; DF = Dilution Factor; RDE=Real-Driving Emissions; WLTC = Worldwide harmonized Light vehicles Test Cycle.

The data needed for the analysis were: idle emissions during cold start (within the first 300 s, but after ignition on >30 s), idling emissions with hot engine (engine on >700 s, typically around 1500 s), PN emissions of type approval cycles (NEDC or WLTC), or RDE compliant tests. Figure 2 gives an example of the first 1500 s of a cold WLTC and an RDE test, in order to explain how the idle data were estimated. The vehicle was a GDI with emissions approximately 3 × 10^12^ p/km.

#### 2.3.1. Idle Concentrations

Starting with the laboratory WLTC test (Figure 2a), idling periods (i.e., speed is 0 km/h and exhaust flow rate >3 kg/h) were at times around 100−120 s, 350−400 s, 1000 s and 1450 s. The “cold” idle concentration levels were considered during the first idle (around 120 s in this example), after the first ignition of the engine (>30 s) and before 300 s (defined as cold start duration in the European regulation). The exclusion of the first 30 s was decided to exclude the very high start-up emissions due to incomplete combustion [47,48]. It also ensured that the start-up emissions with gasoline fuel of CNG and LPG vehicles would not be considered. Furthermore, measuring within 30 s would be very difficult in practice with high risk of damaging the PTI systems due to condensation [49]. The “hot” idle concentrations were determined at periods where the engine was on at least 700 s (e.g., for some NEDCs at 780 s), but usually around 1000 or 1450 s. For the example of Figure 2a, the “hot” idle concentrations were determined at around 1020 and 1470 s. The concentrations were measured with PEMS or PMP systems measuring from the tailpipe (most of the cases with PMP systems). Averages of the last 10 s were used in order to minimize any influence from concentrations of previous engine modes. Data from the dilution tunnel were considered only for a few exceptional cases (7 GDIs and 3 diesel vehicles, all without particle filter) where no tailpipe measurements were available and there was lack of the specific technologies and emission levels, i.e., GDIs emitting >6 × 10^12^ p/km and diesel vehicles without DPF. Figure 2a also shows the concentrations estimated from the PMP system at the dilution tunnel, taking into account the dilution factor (DF) at the dilution tunnel. The DF was calculated by dividing the total dilution tunnel flow to the exhaust flow, typically estimated as the difference between total flow and dilution air flow. While there is a rather good agreement at most parts of the cycle, at idle the deviations are quite high (note the logarithmic scale of the y-axis). One reason is the high uncertainty of the DF determination at idling periods: the exhaust flow is the difference of two large values of similar magnitude. The other is the diffusion that takes place between the vehicle and the dilution tunnel. While at high speeds (and exhaust flow rates) the effect is negligible, at idle, due to the low flow rate and the long residence time, the effect is significant and the concentrations of the section before the idling have a big impact.

Figure 2b plots the same information as in Figure 2a for an RDE trip. The specific RDE trip was not fully compliant with the cold start provisions of the regulation (idling periods), because the test was conducted before the entry into force of the regulation. In this specific example, the “cold” idle concentrations were estimated at 100 s, while the “hot” at 900 and 1400 s. The idle emissions at 900 s were much lower than at 1400 s, nevertheless both values were used for the calculation of the average. The PN concentrations were measured with a PEMS. A similar approach was used for the other vehicles analyzed, depending on the available data.

#### 2.3.2. Type Approval Cycle Emissions

The emission of the WLTC and the RDE tests were calculated according to the equations and the procedures described in the relative regulations (i.e., PMP system at the dilution tunnel for NEDC or WLTC, PEMS at the tailpipe for RDE tests) (Commission Regulation (EU) 2017/1151 and all amendments and corrections). In some cases, laboratory cycles with hot engine start were available. In the text all laboratory cycles (NEDC or WLTC) are with cold engine start, unless specified differently (hot NEDC or hot WLTC).

#### 2.3.3. Measurement Uncertainty

Table 2 summarizes the results of the GDI vehicle presented in Figure 2, where all the information mentioned above was available. However, this was not always true for the rest vehicles, where only limited information was available. Although the repeatability of the emission tests is within acceptable levels (<10%, see last row of Table 2), there are significant differences at the absolute levels between the different cycles and locations. Comparing cold and hot cycles, the cold cycle emissions are almost double for this vehicle (3.6 × 10^12^ p/km vs 1.8 × 10^12^ p/km). The high differences between cold and hot cycles are well known. The enrichment of the air/fuel mixture during cold-start engine operation, in order to compensate for the reduced fuel vaporization and elevated engine components friction, leads to incomplete fuel combustion and higher emissions [50]. A difference of 23% is also noted between tailpipe and dilution tunnel (3.6 × 10^12^ p/km vs 2.9 × 10^12^ p/km) for the cold WLTC. This difference is 11% for the hot WLTC (1.8 × 10^12^ p/km vs 1.6 × 10^12^ p/km). Dedicated studies attributed these differences to exhaust flow uncertainties (for both cold and hot WLTC) and particle agglomeration in the transfer tube to the dilution tunnel especially during cold start (for cold WLTC) [28].

The idle concentrations of a vehicle can have a wide range as well. For the vehicle of Table 2, the “cold” idle concentrations were 17−23 × 10^5^ p/cm^3^, when determined from the tailpipe, but less than half when determined from the dilution tunnel. The hot idle emissions ranged from 1.6 to 6.1 × 10^5^ p/cm^3^, the highest values measured at the RDE tests. For this specific example, the concentration that would be plotted would be the 2.8 × 10^5^ p/cm^3^. The repeatability which was calculated from the particle concentrations (p/cm^3^) at different days varied from 5% to 41% at the laboratory but it was 58% on the road. 

Another example is the emissions of the GDI Golden car of the inter-laboratory exercise with a Golden PN-PEMS [30]. The average laboratory (NEDC) emissions were 1 × 10^12^ p/km with one standard deviation of all laboratories of 24%. The on-road tests had an average of 1.1 × 10^12^ p/km with a similar standard deviation of 25%. The specific uncertainty includes the variability of the vehicle, and the uncertainty of the (same) Golden measurement instrument and exhaust flow meter.

The previous examples give the order of magnitude of uncertainty that the results will have. The PN emissions in one laboratory may have a variability of 10%, but the expected reproducibility levels (i.e., variability between different laboratories) are 20–40% as many studies have shown (see, e.g., reviewed studies in [31]). RDE tests could differ by a factor of two compared to the laboratory type approval cycle. The idle concentrations have a repeatability of 5–60%, but the levels can differ by a factor of 3−4, depending on when they were determined (i.e., engine conditions).

#### 2.3.4. Start and Stop Function and Hybrids

There was one more difficulty in the collection of data. Many vehicles had the start and stop function, so for them there were no idle emissions. For these vehicles, sometimes idle emissions were available at the RDE trips and were used in the included dataset. Only cases where idle emissions could be identified were included in the dataset. 

The hybrid vehicles had a similar difficulty, because the engine was almost always off during idling, regardless of the state of charge of the high voltage battery. For these vehicles, as typically the engine is working in a small operation range of revolutions per minute (rpm), the “idle concentrations” were estimated from the emissions of the vehicle at a constant low speed (below 50 km/h). For hybrid vehicles, the scatter of the WLTC emissions can also vary largely depending on the state of charge of the battery. Here, the emissions at charge sustaining mode were considered, which correspond to the maximum use of the internal combustion engine over the type-approval cycle. The number of hybrid vehicles was low (5) and the emissions also low, below 7 × 10^11^ p/km (except one 1.5 × 10^12^ p/km), so there was no reason to determine more accurately the idle levels.

## 3. Results

### 3.1. Compression Ignition Vehicles

**Figure 3:**
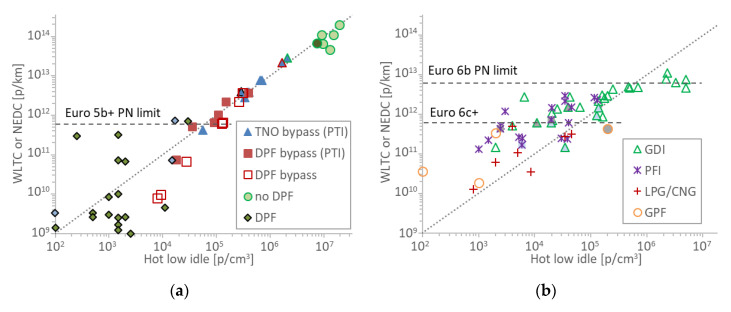
Scatter plots of hot idle concentrations with WLTC (Worldwide harmonized Light vehicles Test Cycle) or NEDC (New European Driving Cycle) type approval test cycles emissions at TNO or JRC (rest points): (**a**) compression ignition vehicles; (**b**) positive ignition vehicles. Each point is a vehicle. Hybrids have grey background. Vehicles with start and stop have light blue background. Horizontal lines give the respective Particle Number (PN) limits. CNG = Compressed Natural Gas; DPF=Diesel Particulate Filter; GDI = Gasoline Direct injection; GPF = Gasoline Particulate Filter; LPG = Liquefied Petroleum Gas; PFI = Port Fuel Injection; PTI = Periodical Technical Inspection; PTI = Periodical Technical Inspection.

Figure 3a illustrates the idle (p/cm^3^) and cycle (p/km) emission results for the compression ignition vehicles. The 1 × 10^7^ cm^3^/km dotted line is also shown as a guide to the eye. The vehicles without DPF have emissions around 10^14^ p/km and the hot idle concentrations are around 10^7^ p/cm^3^. Note that one point with dark green background was a DPF equipped vehicle, with defect or removed DPF. The DPF equipped vehicles have emissions up to 7 × 10^11^ p/km and hot idle concentrations up to 3 × 10^4^ p/cm^3^. The dedicated tests bypassing the DPF (TNO and JRC bypass PTI) fit nicely on the 1 × 10^7^ cm^3^/km (dotted) line. The points “DPF bypass” are the JRC tests bypassing the DPF but determining the hot idle concentrations from the cycle (CVS) and a PMP system instead of a PTI instrument. The idle concentrations differ almost by a factor of two compared to the dedicated test (DPF bypass PTI). The different instruments used, in addition to the procedure itself, have contributed to this difference. The scatter of points around the 1 × 10^7^ cm^3^/km line is very high up to 1 × 10^5^ p/cm^3^, which corresponds to a detection limit of the methodology (i.e., determining high emitter from idle emissions) of 1 × 10^12^ p/km. The Dutch proposed idle limit of 2.5 × 10^5^ p/cm^3^ corresponds approximately to 2.5 × 10^12^ p/km, approximately four times the type approval laboratory limit.

### 3.2. Positive (Spark) Ignition Vehicles

Figure 3b plots the results for the spark (positive) ignition vehicles. The CNG and LPG fueled vehicles have emissions up to 5 × 10^11^ p/km and the hot idle concentrations up to 4.5 × 10^4^ p/cm^3^. The PFI vehicles have emissions up to 3 × 10^12^ p/km and hot idle concentrations up to 1.1 × 10^5^ p/cm^3^. The GDI vehicles have emissions up to 8 × 10^12^ p/km and hot idle concentrations are up to 5 × 10^6^ p/cm^3^. The GPF equipped vehicles have emissions up to 4.3 × 10^11^ p/km and hot idle concentrations up to 2 × 10^5^ p/cm^3^. The GPF vehicle with the highest hot idle emissions was a hybrid, where the idle emissions were determined at a constant speed because the engine was always off at 0 speed. Otherwise the three vehicles propelled by the internal combustion engine fitted with a GPF had hot idle concentrations lower than 3 × 10^3^ p/cm^3^.

The scatter of points around the 1 × 10^7^ cm^3^/km line is very high. Up to 2 × 10^5^ p/cm^3^ idle concentrations there is no correlation between the type-approval emission and the hot idle concentrations, indicating that the limit of detection of the methodology for spark ignition vehicles is 3 × 10^12^ p/km (i.e., below 2 × 10^5^ p/cm^3^ idle concentrations it is not possible to estimate the type-approval emission, which are below 3 × 10^12^ p/km). Interestingly, hot idle concentrations >2.5 × 10^5^ p/cm^3^, which is the proposed idle limit for diesel vehicles, correspond to emissions >4 × 10^12^ p/km. Vehicles with emissions >6 × 10^12^ p/km have idle concentrations >2 × 10^6^ p/cm^3^.

### 3.3. Cold Idle Levels

**Figure 4:**
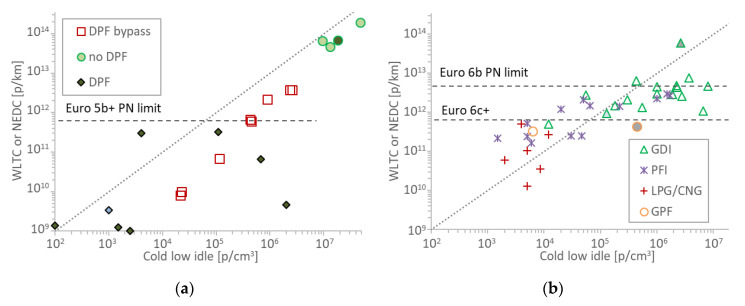
Scatter plots of cold idle concentrations with WLTC (Worldwide harmonized Light vehicles Test Cycle) or NEDC (New European Driving Cycle) type approval test cycles emissions: (**a**) compression ignition vehicles; (**b**) positive ignition vehicles. Each point is a vehicle. Hybrids have grey background. Vehicles with start and stop have light blue background. Horizontal lines give the respective Particle Number (PN) limits. CNG = Compressed Natural Gas; DPF = Diesel Particulate Filter; GDI = Gasoline Direct injection; GPF = Gasoline Particulate Filter; LPG = Liquefied Petroleum Gas; PFI = Port Fuel Injection; PTI = Periodical Technical Inspection.

As cold start emissions constitute a significant portion of the emissions, an effort was made to see whether “cold” idle concentrations correlate better with the type approval cycle values. Figure 4 summarizes the results. The “DPF bypass” and “no DPF” points were taken from the CVS, while the “DPF” points were taken from the tailpipe. 

The points are shifted to the right (i.e., higher idle concentrations) compared to Figure 3 (hot idle), especially for the DPF vehicles. Furthermore, the scatter seems higher due to the high variability of cold start idle concentrations. However, the number of points are less than of Figure 3 and it is difficult to draw a solid conclusion. The cold idle concentrations of the spark ignition vehicles did not have better correlation with the type approval cycles. Only three GDI idle points were taken from CVS and all the rest from the tailpipe. No idle level was found to indicate high emitters. Actually, the correlation got worse. 

### 3.4. Idle and RDE Correlation

**Figure 5:**
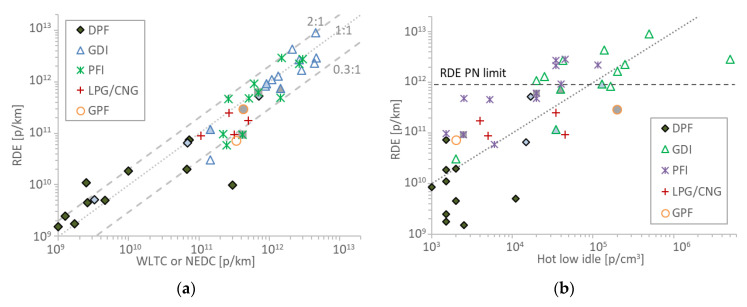
Scatter plots of RDE tests vs.: (**a**) cold start WLTC or NEDC; (**b**) hot idle concentrations. Hybrids have grey background. Vehicles with start and stop have light blue background. CNG=Compressed Natural Gas; DPF = Diesel Particulate Filter; GDI = Gasoline Direct injection; GPF = Gasoline Particulate Filter; LPG = Liquefied Petroleum Gas; PFI = Port Fuel Injection; PTI = Periodical Technical Inspection.

Another question is how representative the idle—type-approval cycle correlation is when vehicles are driven on the road. Figure 5a compares WLTC or NEDC with RDE tests for vehicles for which both tests were available. For the vehicles tested in this study the RDE tests were between 3 times lower and two times higher than the corresponding WLTC results. This variability is higher than the maximum measurement expected uncertainty of the PEMS (sensor plus exhaust flow meter) which is 50% [9]. There was no particular trend of a specific technology or fuel for larger or smaller differences between laboratory and on-road results. Figure 5b shows hot idle concentrations versus the RDE emissions. The results are quite similar to the idle-WLTC/NEDC scatter plot (Figure 3) and they indicate that the idle and type approval cycle correlation is a good indication of the real world behavior of the vehicle. However, there are only three points at or above 2.5 × 10^5^ p/cm^3^: one having emissions around 2.4 × 10^12^ p/km (idle 2.5 × 10^5^ p/cm^3^), the other 9.1 × 10^12^ p/km (idle 5 × 10^5^ p/cm^3^), and the last one 2.9 × 10^12^ p/km (idle 5 × 10^6^ p/cm^3^). The last point is not in agreement with the idle and type approval cycle correlation where idle concentrations above 2 × 10^5^ p/cm^3^ corresponded to emissions >4 × 10^12^ p/km, but it’s quite near (2.9 × 10^12^ p/km). Thus, more studies correlating RDE and idle concentrations are necessary for high emitting vehicles.

## 4. Discussion

The aim of this study was to compare PN concentrations at low idle with PN emissions of type approval cycles (WLTC or NEDC). The only dedicated tests (i.e., type approval test and then hot idle test) were taken from the literature. The remaining data points were based on averages of hot idle concentrations measured during the laboratory or on-road tests.

This approach had some challenges. First of all, the idle PN concentrations were not constant throughout the test. Typically, during cold start, higher concentrations were measured. But, even with hot engine the levels varied significantly. A factor of 3–4 as variability was sometimes seen, and the repeatability (i.e., the idle concentrations at the same time period over different days) was not so good (<60%). Similar behavior has been seen in other studies and has been attributed to the different exhaust gas recirculation (EGR) percentage [22]. Many vehicles utilized start and stop, so it was challenging to find the idle concentrations. Moreover, the hybrid vehicles switched the engine off when the speed was zero. The laboratory type approval tests had much better repeatability (10%), but reproducibility levels of 20–40% are common for PN measurements at different laboratories [31,51]. 

### 4.1. Limit of Detection of PTI Methodology

#### 4.1.1. Compression Ignition Vehicles

The results of the compression ignition vehicles showed that the correlation of idle concentrations and type approval cycle emissions is quite good for idle concentrations >1 × 10^5^ p/cm^3^ and emission levels >1 × 10^12^ p/km. Below these levels the data had a high scatter. This finding is important because it demonstrates that the limit of detection of the PTI sensors does not need to be too low and even 2.5 × 10^4^ p/cm^3^ (10% of the limit), which is the minimum required accuracy in the Dutch regulation, is more than enough.

#### 4.1.2. Spark Ignition Vehicles

The results of the positive (spark) ignition vehicles did not have a good correlation, as the scatter was very high. Nevertheless, the results indicated that the limit of detection of the methodology is around 3 × 10^12^ p/km. Hot idle concentrations of >2.5 × 10^5^ p/cm^3^ corresponded to >4 × 10^12^ p/km emission levels. These results are very encouraging as the same limit for diesel vehicles (2.5 × 10^5^ p/cm^3^) may be applicable to spark ignition vehicles. For high emitting GDIs (i.e., >6 × 10^12^ p/km), the idle emissions were >2 × 10^6^ p/cm^3^. However, the idle levels of the high emitting GDI vehicles were determined from the CVS, thus they could be different compared to direct tailpipe measurements, and further studies are necessary to confirm these results.

The correlation did not improve (actually got worse) when the cold idle concentrations were used. Thus, it is not necessary to define a cold test for PTI testing, which is also impractical as most of the times vehicles undergo PTI in warm conditions. As mentioned in the Dutch regulation, the test can be done with cold engine, but if it fails, it should be repeated with warm engine.

The results are in agreement and further expand the findings of a previous study on GDIs [22]: the hot idle concentrations of the tested GDI vehicles with GPF (three Euro 6d-temp) were 0.1−1.2 × 10^4^ p/cm^3^, while for those without GPF (three Euro 5 or Euro 6b) were 5–10 × 10^4^ p/cm^3^. The pre-GPF emissions of the Euro 6d-temp GDIs were high: 0.1–5.8 × 10^6^ p/cm^3^. Combining these results with ours, it seems that GPF equipped vehicles have similar behavior with DPF equipped diesel vehicles: the idle concentrations are lower than 2–3 × 10^4^ p/cm^3^ (exception the hybrid GPF vehicle). In most cases, GDI vehicles without GPF have higher idle concentrations. Thus, identifying tampering of the GPF may be possible. However, this needs more research because there are already GDI vehicles without GPF having low idle concentrations and type-approval emissions. In addition, idle levels of 10^4^ p/cm^3^ to 2 ×10^5^ p/cm^3^ correspond to relatively low emissions (6 × 10^11^ p/km to 2 × 10^12^ p/km).

#### 4.1.3. Reasons of High Scatter

As already indicated, while for compression ignition (diesel) engines, the hot idle emissions had a relative good correlation with the type approval cycle, this was not the case for the positive (spark) ignition engines. In diesel engines fuel is injected into the engine cylinder and mixes with high temperature-pressure air [52]. As the piston moves to the top-dead-center, the mixture reaches the ignition point and the combustion of the charge starts as premixed combustion and continues as diffusion limited combustion. Although the overall air-to-fuel ratio is lean, combustion occurs when vaporized fuel mixes with air stoichiometrically. Soot emissions are generally formed at the fuel-rich side of the reaction zone in the diffusion combustion phase [53]. Diesel combustion is heterogeneous in nature, compared to spark ignition engines in which the combustible mixture is predominantly homogenous. In conventional PFI engines, fuel is injected into the intake port so that fuel and air flow simultaneously into the combustion chamber during the intake process, and a homogeneous air–fuel mixture is formed. PFI engines have low emissions at steady state conditions or light loads. Spikes of PN are seen when driving requires fuel enrichment, such as cold start, accelerations and high loads [54]. As the majority of the emissions originates from these events, the idle concentrations do not necessarily correlate with the overall cycle emissions. In GDI engines, fuel is sprayed directly into the combustion chamber. This leads to incomplete fuel evaporation due to the limited time available for fuel and air mixing, resulting in localized rich combustion and PM formation [55]. Additionally, a small amount of fuel may impinge on the piston and make direct contact with the cold cylinder walls, which may lead to diffusion combustion and subsequent PM formation [56], in particular during fuel enrichment events. Thus, it seems that the idle concentrations cannot “represent” the fuel enrichment events of the GDI engines.

The even higher scatter of vehicles equipped with particulate filters (DPF, GPF) can be explained by the different mechanism of particles’ appearance. Particulate filters are generally very efficient in removing particles, with filtration efficiencies >95% [52,57]. Nonetheless, in some cases, high particle concentrations are measured during cold start [8]. The main reason for these emissions are small defects in the mat used to mount the brick in the canister, resulting in reduced filtration efficiency [58]. The defects close as the particulate filter heats up and the filtration increases. The second issue with filters is that the filtration efficiency depends on the accumulated soot and deposited ash. For example, emissions are very high immediately after a regeneration event, but drop significantly only after a few minutes of driving [8]. Similarly, over time, due to deposited ash that cannot be burnt during regenerations, the filtration efficiency on average improves. In particular for GPFs, filtration improvements of 10–15% after only 3000 km have been reported [59]. It is expected that a normal use will cover more than 3000 km in four years when the first PTI will take place.

### 4.2. DPF Soot Load and Idle Levels

Table 3 summarizes the WLTC idle concentrations of three DPF equipped vehicles just before and immediately after regeneration events. The cold idle concentrations were determined at 120 s, while the hot idle concentrations at 1000 s. Immediately after regeneration, the cold idle exceeded the limit of 2.5 × 10^5^ p/cm^3^ for two of the vehicles. However, the hot idle concentrations were <3 × 10^3^ p/cm^3^ for all three vehicles. Thus, driving 10–15 min after a regeneration event should be sufficient to form a soot cake at the DPF and drop the idle concentrations at the typical levels for the specific vehicle. 

### 4.3. Implications for PTI Sensors

The results section was based on PEMS and PMP systems. PMP systems typically weight >50 kg and are fixed in the laboratory. PN-PEMS typically weight >10 kg and can easily fit in the trunk or on the hook of a vehicle. Both instruments are handled by experienced and specialized personnel. PTI instruments should be handheld in a garage type of environment and therefore they have to be robust and easy to use. Sensors that weight only 0.4 kg have been reported, however the prototype instruments are much heavier [60]. Obviously, their technical specifications cannot be as strict as of those of PEMS or PMP systems. Table 4 summarizes the efficiency (i.e., ratio to a reference system) requirements of PEMS and PMP systems [8,9], along with those of PTI sensors based on the Dutch legislation [26], and the Swiss regulation for construction machinery [61]. The Dutch regulation can be considered representative of future PTI regulations for diesel cars of other countries. The efficiency requirements are very similar for all regulations with only minor differences (e.g., calibration size and polydispersity for the Swiss regulation). The required accuracy is around 30% at large sizes (efficiency 0.70 to 1.30). The Dutch regulation requires maximum measurement error of 25% or 2.5 × 10^4^ p/cm^3^ (whichever is larger). Although the 2.5 × 10^4^ p/cm^3^ concentration is higher than the 5 × 10^3^ p/cm^3^ zero level required for PEMS, it is sufficient, as it was shown in the results section. The maximum concentration requested for these sensors is 5 × 10^6^ p/cm^3^. This concentration is appropriate for DPF equipped vehicles, even with cracks or partial damage, but not for older diesel vehicles without DPF.

At 23 nm the required efficiency is around 40% (± 20%). The requirement is quite similar to the estimated efficiency of the PMP systems (33–60%). The 23 nm size was selected by the PMP group in order to include the smaller soot particles, but at the same time exclude any nucleation mode volatile particles [8]. The PEMS and PTI requirements for the steepness of the cut-off curve are not as strict as for the PMP system. Thus, particles <23 nm may be counted. This should not be an issue for diesel vehicles, for which the majority of particles are >23 nm (e.g., [7]). The only exception is during cold start where high concentrations of nonvolatile sub-23 nm particles at idle or low speeds can be seen [42,62,63]. At hot idle the nonvolatile (solid) sub-23 nm particles are absent [63]. For spark ignition vehicles though the sub-23 nm particles can be as many as those >23 nm [7,47,64].

The efficiency requirements of PMP systems can be achieved only by condensation particle counters (CPCs) [65], while those of PEMS or PTI regulations with both CPCs and advanced diffusion charging counters [9,66,67]. Opacimeters or light scattering instruments would fail these specifications.

The volatile removal efficiency requirements of the different regulations are summarized in Table 5. The new PTI requirements are similar to the PMP requirements for systems measuring >23 nm (>99% of 30 nm tetracontane particle). The PTI requirement is much easier than the one for PEMS (mass >1 mg/m^3^), yet as the lower cut-off diameter is at 23 nm the evaporation efficiency is sufficient. Diesel vehicles have high air-fuel ratio and condensation is unlikely. Volatile nucleation mode particles can be seen with high exhaust gas temperature and high sulfur content [68], but at other conditions are less likely. For spark ignition engines nucleation particles at low idle are also not probable [69]. However, volatile compounds can grow the sub-23 nm solid particles in the >23 nm range, affecting the results and the comparability between different instruments. Thus, some thermal pre-treatment is necessary. Theoretically, the tetracontane removal requirement is easily achievable by heating the aerosol at 200 °C [70]. Discussion on the thermal pre-treatment topic can be found elsewhere [57,71,72]. Commercial systems use catalytic stripper, sensors at elevated temperature or include hot dilution (see detailed discussion of available equipment elsewhere [16]).

Currently, the instruments used for PTI testing are prototypes and similar to the PEMS [17]. Thus, their measurement uncertainty is around 35% and compared to the PMP systems at the full dilution tunnel the differences are around 50% [9]. Other smaller PTI sensors had differences by a factor of two during a measurement campaign [29]. Thus, it is necessary to further characterize the instruments that will appear in the market in the future.

All results and the discussion so far focused on the current regulation which counts nonvolatile particles >23 nm. Recently, in the Global Technical Regulation (GTR 15) of the worldwide harmonized light vehicles test procedure (WLTP), the new proposal includes counting solid particles >10 nm [73]. For countries adopting this option, the new PTI procedures should be adopted accordingly. As discussed previously, for diesel vehicles the effect should be minimal (other than changing the cut-off size of the instruments), however for spark ignition vehicles further studies are needed. For example, high concentrations of sub-23 nm solid particles have been reported for CNG and PFI vehicles (see, e.g., [7,64]). For filtered equipped vehicles the effect should be small because sub-23 nm particles should be captured with high efficiency, but for faulty filters of spark ignition vehicles high concentration of both >23 nm and sub-23 nm particles will pass through the faulty filter and this needs to be examined.

## 5. Conclusions

The new periodical technical inspection (PTI) procedure will require measurement of vehicle exhaust particle number (PN) concentrations at idle. In this study the PN emissions of type approval cycles were compared with low idle concentrations for diesel, gasoline (GDI or PFI), CNG and LPG vehicles. For diesel vehicles the correlation was good for PN levels >1 × 10^12^ p/km (idle concentration >1 × 10^5^ p/cm^3^). At lower emission levels the cycle emissions depended significantly on the cold start emissions, due to the existence of DPFs. However, the correlation did not improve correlating the emissions with the cold idle concentrations.

For positive (spark) ignition vehicles, no correlation between cycle emissions and idle concentrations could be found. For this category of vehicles, the emissions are mainly produced during fuel enrichments, e.g., during cold start, accelerations, or high loads. Nevertheless, idle concentrations >2.5 × 10^5^ p/cm^3^ were related to emission levels >4 × 10^12^ p/km and idle concentrations >2 × 10^6^ p/cm^3^ were related to emission levels >6 × 10^12^ p/km. Although these levels are a first step in defining limits for this category of vehicles, more dedicated studies are necessary, especially if a correlation against RDE is also of interest. Further tests are also needed for hybrid vehicles. 

The current PTI technical specifications are comparable to those of on-board and laboratory type-approval systems and of sufficient stringency. The current PTI systems are similar to the on-board systems. However, as they will become smaller in size and less complex, more tests with dedicated PTI sensors are also important in order to assess their measurement uncertainty. 

## Figures and Tables

**Table 1:** Summary of available vehicles. Number after “+” are vehicles from literature (dedicated new PTI test).

	WLTC	NEDC	RDE	Cold Idle	Hot Idle	Comment
Diesel w/o DPF	0	6 ^1^	0	5	6	3 from CVS
Diesel with DPF	18 + 2	2 + 4	15	8	26	
GDI w/o GPF	17 + 1	9	13	17	27	6 from CVS
GDI with GPF	3 + 1	0	2	2	4	
PFI	14	4	13	13	18	
LPG or CNG	7	0	4	6	7	

^1^ two of them L-category mini cars CNG = Compressed Natural Gas; CVS = Constant Volume Sampling; DPF = Diesel Particulate Filter; GDI = Gasoline Direct injection; GPF = Gasoline Particulate Filter; LPG = Liquefied Petroleum Gas; NEDC = New European Driving Cycle; PFI = Port Fuel Injection; PTI = Periodical Technical Inspection; WLTC = Worldwide harmonized Light Vehicles Test Cycle.

**Table 2:** Results for the vehicle presented in Figure 2. Numbers in brackets give one standard deviation or half of max-min when two repetitions were available. Number of repetitions (n): n = 4 for cold WLTC and RDE, n = 2 for hot WLTC. CVS = Constant Volume Sampling (dilution tunnel); RDE = Real-Driving Emissions test; WLTC = Worldwide harmonized Light vehicles Test Cycle.

	Cold WLTC CVS	Cold WLTC Tailpipe	Hot WLTC CVS	Hot WLTC Tailpipe	RDE ^1^ Tailpipe
Cold idle [p/cm^3^]	8.6 × 10^5^ (42%)	17.1 × 10^5^ (10%)	-	-	23.4 × 10^5^ (32%)
Hot idle [p/cm^3^]	4.8 × 10^5^ (41%)	2.8 × 10^5^ (4%)	3.9 × 10^5^ (5%)	1.6 × 10^5^ (16%)	6.1 × 10^5^ (58%)
Cycle emissions [p/km]	2.9 × 10^12^ (±5%)	3.6 × 10^12^ (±7%)	1.6 × 10^12^ (±7%)	1.8 × 10^12^ (±6%)	1.8 × 10^12^ (±8%)

^1^ Not fully compliant with RDE regulation regarding idling cold start provisions.

**Table 3:** Particle number (PN) cycle emissions and cold idle (at 120 s) and hot idle (at 1000 s) concentrations for WLTCs (Worldwide harmonized Light vehicles Test Cycle) just before and immediately after regenerations.

Vehicle	PN [p/km]	Cold Idle [p/cm^3^]	Hot Idle [p/cm^3^]	Study
DPF #1 before	2.6 × 10^9^	n/a	<1.0 × 10^3^	[34]
DPF #1 after	2.0 × 10^11^	2.8 × 10^5^	1.0 × 10^3^	[34]
DPF #2 before	3.0 × 10^9^	<1.0 × 10^3^	<1.0 × 10^3^	[35]
DPF #2 after	9.5 × 10^11^	1.5 × 10^6^	2.0 × 10^3^	[35]
DPF #3 before ^1^	2.0 × 10^9^	n/a	1.0 × 10^3^	[42]
DPF #3 after ^1^	6.0 × 10^10^	1.8 × 10^4^	2.7 × 10^3^	[42]

^1^ Measured from the dilution tunnel.

**Table 4:** Efficiency requirements for various systems using monodisperse (mono) or polydisperse (poly) aerosol.

Diameter	Aerosol	23 nm	30 nm	50 nm	70−100 nm	200 nm
PMP ^1^	Mono	0.33–0.60	0.59–0.91	0.99–1.00	1.00–1.13	1.00–1.14
PEMS ^2^	Mono	0.20–0.60	0.30–1.20	0.60–1.30	0.70–1.30	0.50–2.00
**Diameter**	**Aerosol**	**23 nm**	**41 nm**	**50 nm**	**80 nm**	**200 nm**
Dutch PTI ^3^	Mono	0.20–0.60	-	0.60 – 1.30	0.70–1.30	-
Swiss PTI ^4^	Poly	<0.50	>0.40	-	0.70–1.30	<3.00

^1^ Estimated from Regulation (EU) 2017/1151 requirements for separate parts; ^2^ Regulation (EU) 2017/1154; ^3^ Dutch regulation No. IENW / BSK-2019/202498 [26]; ^4^ Swiss regulation for construction machinery VAMV SR 941.242 [61]. PMP = Particle Measurement Programme; PEMS = Portable Emissions Measurement Systems; PTI = Periodical Technical Inspection; VAMV = Ordinance of the FDJP on Exhaust Gas Analyzers.

**Table 5:** Volatile removal efficiency requirements of tetracontane particles for various systems.

Diameter	Aerosol	Diameter	Number Conc.	Mass Conc.	Efficiency
PMP ^1^	Mono or poly	≥30 nm	≥10^4^ p/cm^3^	-	≥99%
PEMS ^2^	Poly	≥50 nm	-	≥1 mg/m^3^	≥99%
Dutch PTI ^3^	Mono	≥30	0.5−1 × 10^4^ p/cm^3^	-	≥95%
Swiss PTI ^4^	Poly	≥30	<10^5^ p/cm^3^	-	≥95%

^1^ Regulation (EU) 2017/1151; ^2^ Regulation (EU) 2017/1154; ^3^ Dutch regulation No. IENW / BSK-2019/202498 [26]; ^4^ Swiss regulation for construction machinery VAMV 941.242 [61]. PMP = Particle Measurement Programme; PEMS = Portable Emissions Measurement Systems; PTI = Periodical Technical Inspection; VAMV = Ordinance of the FDJP on Exhaust Gas Analyzers.
